# Supplementary material for: Content-rich biological network constructed by mining PubMed abstracts
Source: BMC Bioinformatics. 2004 Oct 8;5:147. doi: 10.1186/1471-2105-5-147 (PMC528731; doi:10.1186/1471-2105-5-147)
Supplement: Additional File 2 — The original results of the above study (non-essential files are deleted to keep the file size under the limit set by BMC bioinformatics). [file 1471-2105-5-147-S2.bz2 › chilibotAdditionalFile2/dip05/185ID7526154E627/html/IFNAR1_TYK2.html]

 


 **IFNAR1** and **TYK2** 
  
Found 47 abstracts in PubMed, retrieved 05.  
 

 What does Google say? 
 PDF only 
| .edu only 

---

**Interactive relationship** (e.g. stimulation, inhibition, etc)

**Stimulatory relationship**- Conversely, co expressed  **Tyk2**  greatly enhances surface  **IFNAR1**  expression.  Ref: 12554654 EMBO J, 2003
- AVP  [ **IFNAR1** ]  also stimulates the tyrosine phosphorylation of the nonreceptor tyrosine kinase proline rich tyrosine kinase 2  [ **TYK2** ]  Pyk2 and promotes Src family kinase phosphorylation at Tyr 418, indicative of Src activation.  Ref: 11832328 Am J Physiol Cell Physiol, 2002
**Neutral relationship**- The tyrosine kinase  **Tyk2**  controls  **IFNAR1**  cell surface expression.  Ref: 12554654 EMBO J, 2003
- Importantly, we demonstrate that  **Tyk2**  slows down  **IFNAR1**  degradation.  Ref: 12554654 EMBO J, 2003
- In the type I interferon receptor,  **Tyk2**  associates with the  **IFNAR1**  receptor subunit and positively influences ligand binding to the receptor complex.  Ref: 12554654 EMBO J, 2003
- Here, we report that  **Tyk2**  is essential for stable cell surface expression of  **IFNAR1** .  Ref: 12554654 EMBO J, 2003
- However, a dose dependent interference with signal transduction via the endogenous Ifnar  [ **IFNAR1** ]  complex was found for STAT1, STAT2, STAT3,  **Tyk2** , and Jak1 activation, for gene induction, and for antiviral activity.  Ref: 11602573 J Biol Chem, 2001

**Non-interactive relationship** (e.g. studied together, co-existance, homology, etc.)

- In the absence of  **Tyk2** , mature  **IFNAR1**  is weakly expressed on the cell surface.  Ref: 12554654 EMBO J, 2003
- In both model systems, titration of the  **Tyk2**  kinase away from the  **Ifnar1**  receptor chain accounts for the observed cross interference.  Ref: 11602573 J Biol Chem, 2001
- In murine macrophages, IFN beta induced TLR3 up regulation required  **IFNAR1** , STAT1, and in part IRF 1, but not the Janus kinase Jak family member  **Tyk2** .  Ref: 12672806 J Biol Chem< MedlineTA>J Biol Chem, 2001
- Despite  **Tyk2**  activation, erythropoietin treatment of cells expressing this erythropoietin receptor  **Ifnar1**  chimera did not evoke any detectable IFN type response.  Ref: 11602573 J Biol Chem, 2001
